# The Tanzania Field Epidemiology and Laboratory Training Program: building and transforming the public health workforce

**Published:** 2011-12-14

**Authors:** Peter Mmbuji, David Mukanga, Janeth Mghamba, Mohamed Ahly, Fausta Mosha, Simba Azima, Sembuche Senga, Candida Moshiro, Innocent Semali, Italia Rolle, Stefan Wiktor, Suzzane McQueen, Peter McElroy, Peter Nsubuga

**Affiliations:** 1 Tanzania Ministry of Health and Social Welfare, P.O. Box 9083, Dar es Salam, Tanzania; 2 African Field Epidemiology Network P.O. Box 12874, Kampala, Uganda; 3 Muhimbili University of Health and Allied Sciences, P.O. Box 65001, Dar es Salaam, Tanzania; 4 National Institute for Medical Research, P.O. Box 9653, Dar es Salaam, Tanzania; 5 Centers for Disease Control and Prevention, Tanzania; 6 Center for Global Health, Centers for Disease Control and Prevention, Atlanta Georgia, USA; 7 President's Malaria Initiative, Tanzania

**Keywords:** Field Epidemiology Laboratory Training Program, Tanzania, health workforce, Integrated disease surveillance and response, International Health Regulations, Tanzania

## Abstract

The Tanzania Field Epidemiology and Laboratory Training Program (TFELTP) was established in 2008 as a partnership among the Ministry of Health and Social Welfare (MOHSW), Muhimbili University of Health and Allied Sciences, National Institute for Medical Research, and local and international partners. TFELTP was established to strengthen the capacity of MOHSW to conduct public health surveillance and response, manage national disease control and prevention programs, and to enhance public health laboratory support for surveillance, diagnosis, treatment and disease monitoring. TFELTP is a 2-year full-time training program with approximately 25% time spent in class, and 75% in the field. TFELTP offers two tracks leading to an MSc degree in either Applied Epidemiology or, Epidemiology and Laboratory Management. Since 2008, the program has enrolled a total of 33 trainees (23 males, 10 females). Of these, 11 were enrolled in 2008 and 100% graduated in 2010. All 11 graduates of cohort 1 are currently employed in public health positions within the country. Demand for the program as measured by the number of applicants has grown from 28 in 2008 to 56 in 2011. While training the public health leaders of the country, TFELTP has also provided essential service to the country in responding to high-profile disease outbreaks, and evaluating and improving its public health surveillance systems and diseases control programs. TFELTP was involved in the country assessment of the revised International Health Regulations (IHR) core capabilities, development of the Tanzania IHR plan, and incorporation of IHR into the revised Tanzania Integrated Disease Surveillance and Response (IDSR) guidelines. TFELTP is training a competent core group of public health leaders for Tanzania, as well as providing much needed service to the MOHSW in the areas of routine surveillance, outbreak detection and response, and disease program management. However, the immediate challenges that the program must address include development of a full range of in-country teaching capacity for the program, as well as a career path for graduates.

## Introduction

In 1998 Tanzania was the first country in Africa to launch the World Health Organization, Regional Office for Africa (WHO-AFRO)'s Integrated Disease Surveillance and Response (IDSR) strategy. An assessment of the strengths and weaknesses of the existing public health surveillance and response systems was conducted, and a plan to improve and integrate these systems was developed [1]. Following this assessment, it was recognised that a competently trained public health workforce was needed to operate the public health surveillance and response systems. The Tanzania Ministry of Health and Social Welfare (MOHSW), WHO, the United States (US) Agency for International Development (USAID) and the US Centers for Disease Control and Prevention (CDC) collaborated through a series of initiatives to strengthen the public health workforce in order to implement IDSR in Tanzania [2]. In 2001, through a USAID/MOHSW supported program, an IDSR implementation team comprising the National Institute for Medical Research (NIMR), Partners for Health Reform (PHRplus), Center for Health and Gender Equity (CHANGE) and CDC was tasked by the MOHSW to implement and improve IDSR functioning in 12 districts as pilot [3,4].

Plans to establish a field epidemiology and laboratory training program (FELTP) in Tanzania were conceived in 2005 by the MOHSW, NIMR and CDC. At this time, three trainees from Tanzania were enrolled in the Kenya FELTP [5] with the plan to have them serve as a foundation to establish a program in Tanzania and to improve IDSR implementation. Subsequently, a working group consisting of representatives of the MOHSW, NIMR WHO, and CDC convened to further develop the plan.

Upon a MOHSW request in 2007, an assessment led by the CDC and the African Field Epidemiology Network (AFENET) was undertaken to identify the country's needs, describe the existing infrastructure and resources that could be leveraged to support the program, and define the elements and resources needed to implement a FELTP in Tanzania.

The Tanzania FELTP (TFELTP) was established in October 2008 as a partnership among MOHSW, the Muhimbili University of Health and Allied Sciences (MUHAS), and NIMR, along with support of local and international partners, principally, AFENET, CDC, USAID, and WHO. The goals of the TFELTP are to strengthen MOHSW capacity to i) conduct public health surveillance and response through IDSR, ii) manage and evaluate national disease control and prevention programs, and iii) strengthen public health laboratory support for surveillance, diagnosis, treatment, and clinical monitoring.

## Country context

Tanzania continues to experience a high burden of endemic diseases, as well as recurrent outbreaks of epidemic prone diseases, including cholera, bacterial meningitis, measles, and Rift Valley Fever [6–10]. Successful implementation of IDSR in Tanzania required a competently trained public health workforce comprised of field epidemiologists working in coordination with public health laboratory scientists with the support of a networked system of functional reliable public health laboratories. Since 1998, MOHSW has been training frontline health workers in IDSR through short courses on surveillance, outbreak investigation and response. While this provided an introduction and orientation to key epidemiologic principles and skills, this training in itself was insufficient to maintain the required level of epidemiologic capacity. In addition to increasing the value placed on quality data and the ability to collect and use quality data at the facility and district level, the MOHSW determined there was still a need for intensive epidemiology training to produce a cadre of field epidemiologists and public health laboratory managers to lead IDSR implementation at the regional and national levels.

Tanzania faces a shortage of qualified field epidemiologists at all levels. Similar needs exist for public health laboratorians trained in field epidemiology to be effective members of surveillance and outbreak investigation teams at the national and regional levels. Several factors can be attributed to this, including shortage of relevant training facilities [11]. The MOHSW, in its National Health Policy (2007) [12] and its Human Resource for Health Strategic Plan 2008-2013[13], has emphasized the need to “move towards self sufficiency in manpower by training all the cadres required at all levels from village to national level.” To supplement the short-term training provided by the IDSR strategy, the MOHSW worked with partners including CDC and AFENET to develop and sustain a 2-year FELTP program to train public health professionals in applied epidemiology and laboratory management. These staff would be available as national and regional level resource persons to support districts’ epidemiological needs.

Before TFELTP was established, there were three master-level courses addressing public health issues, a Master of Public Health (MPH), Master of Arts in Health Policy and Management, and a Master of Science (MSc) in Tropical Disease Control in Tanzania. However, MOHSW recognized that in order to achieve its vision of addressing the public health concerns of the population, the number and type of graduates produced at the time was inadequate to meet the growing needs of the country. The TFELTP will facilitate Tanzania's target of training at least 200 epidemiologists and 100 public health scientists as outlined in its Health Sector Strategic Plan III [14].

### Location and structure of the program

The TFELTP program is housed within NIMR headquarters in Dar-es-Salaam, with additional office space for coordinating field activities being at the MOHSW building (Epidemiology and Disease Control Section). TFELTP is a 2-year full-time program involving approximately 25% class room contact, and 75% field placement. Didactic sessions are held within the NIMR building, and are facilitated by MUHAS, MoHSW and NIMR staff. Classroom time occurs across the two years and is interspersed with field placements and examinations. Residents are attached to field placements at the national level in the first year of training and then the regional level during the second year. During the field placement, residents are assigned to various positions to provide epidemiologic and laboratory service to the MOHSW under the supervision of a mentor. The trainees travel to various regions of Tanzania for supervised outbreak investigations and field projects.

### Tracks offered and courses delivered

TFELTP offers two tracks, leading to either an MSc in Applied Epidemiology (Epi Track), or an MSc in Epidemiology and Laboratory Management (Lab Track). The degrees are awarded by MUHAS. Table 1 shows the courses offered within each track by semester.

**Table 1:** Tanzania Field Epidemiology and Laboratory Training Program (TFELTP) courses by track

Semester	Courses	Track
I	Epidemiology and Biostatistics	Both
I	Field Epidemiology and Public Health Surveillance	Both
I	Research Methodology and Computers in Public Health	Both
I	Bioethics	Both
		
II	Advanced Epidemiology	Epi
II	Fundamental Laboratory Methods	Lab
II	Educational Principles and Practices for the Health Sciences Professionals	Both
II	Dissertation: Proposal development and ethical approval	Both
		
III	Economic analysis and evaluation	Epi
III	Laboratory Management, Policy and System design	Lab
III	Dissertation: Data collection	Both
		
IV	Management and Leadership	Both
IV	Dissertation: Data analysis, report writing, examination and dissemination	Both

### TFELTP entry requirements

The entry requirement for the MSc in Applied Epidemiology is a first degree (Bachelor's level) in any of the fields of Medicine, Dentistry, Laboratory Sciences, Veterinary Medicine, Pharmacy, Environmental Health Sciences, Nursing or any other health-related field of study. Other eligible disciplines include statistics, demography, biology, food science and public health. The entry requirement for the MSc in Epidemiology and Laboratory Management is a first degree in the field of Laboratory Sciences, Medical Sciences, Veterinary Medicine, Biological sciences or any other laboratory-related field of study. Applicants for both tracks should have a minimum of two years health related working experience within a government department.

### Other courses offered by TEFLTP

TFELTP conducts a number of short courses that are offered to health workers in Tanzania. Program trainees participate in some of these courses. The courses include: three-month short course in basic field epidemiology and outbreak investigation. Two weeks for didactics at the beginning, with remaining period for an applied field project; three-month short course in non-communicable disease (NCD) epidemiology and prevention. Two weeks for didactics at the beginning, with remaining period for an applied field project.

### Roles of partnerships within TFELTP

The program is governed by a Steering Committee comprising all partners and stakeholders under the leadership of the Chief Medical Officer of the MOHSW. The MOHSW provides the overall leadership for the program including the day to day running of the program through the secretariat. The secretariat is comprised of three ministry of health staff working along with administrative support staff, and program resident advisors.

MUHAS is the primary academic partner for TFELTP. TFELTP courses draw multidisciplinary expertise from within and outside the MUHAS. The university assures the qualification of teaching staff, provides faculty, organizes the assignment of academic supervisors for the residents’ thesis research, and awards the degree upon successful completion of the program. Additionally, MUHAS and program staff teach academic courses and oversee research activities for the students and provide expertise in abstract and manuscript writing.

NIMR provides the base of operations for the FELTP, including office space and training rooms. NIMR staff also ssist with the academic and technical supervision of residents. NIMR through its centers and stations across the country provides facilities for laboratory, data management, and field site opportunities for the trainees.

WHO provides technical and financial support to the program in areas of outbreak investigation and response, and in collaboration with program staff participate in the planning for outbreak and response activities that address the needs of Tanzania.

CDC Tanzania and the programs it represents are the primary funding partners of the TFELTP through the Global AIDS Program (GAP). Additional support is provided by USAID through the President's Malaria Initiative (PMI). CDC Tanzania is the primary link to the MOHSW, MUHAS, and other partners in Tanzania. CDC Tanzania staffs also provide technical assistance through teaching and mentorship of trainees, as well as arranging opportunities for field work including evaluations and field investigations within its programmatic activities in Tanzania.

CDC Atlanta through its Division of Public Health Systems and Workforce Development (DPHSWD) is the partner primarily responsible for providing technical assistance to the FELTP for training and IDSR implementation. With a 30 year history of implementing applied epidemiology training programs in dozens of countries, DPHSWD uses their experienced staff and links to other parts of the agency and partners to build the program. For the initial 3 years of the program DPHSWD managed the AFENET cooperative agreement through which the majority of TFELTP funding was channelled to conduct the day to day operations. CDC technical assistance included teaching, mentoring, and was also directed towards production and dissemination of IDSR bulletins.

AFENET was involved in areas that assisted in preparing the country for the program including, disease surveillance and outbreak response short courses, identification of key partners for the program, and fundraising. AFENET provides technical assistance to TFELTP through teaching, and links TFELTP to other similar program across Africa, providing a platform for exchange of expertise and networking. AFENET is the primary partner responsible for the administrative and logistical implementation of the program.

### Other partners in the program

The World Bank through the East Africa Public Health Laboratory Network is providing support to the program for two slots in 2011-2012, as well as short courses in epidemiology and laboratory management. The International Association of National Public Health Institute (IANPHI) provides support for NCD field sites and has provided 2 slots in 2010-2012. Ifakara Health Institute provides field sites to the program, and its staff for mentorship. TFELTP is currently working with NIMR and Georgia State University to establish an NCD short course on research.

## Highlights and achievements of the program

### Enrolment

Since 2008, the program has enrolled a total of 33 (23 males, 10 females) trainees. Of these, 11 were enrolled in 2008 and graduated in 2010 representing a completion rate of 100%. The 2009 cohort of 10 is expected to graduate at the end of 2011. Demand for the program as measured by the number of applicants has grown from 28 in 2008, to 40 in 2010, to 56 in 2011.

### Employment of graduates

All 11 graduates of cohort 1 have since found public health related employment within the country. Their distribution is as follows: one with a university, two at the National level of the MOHSW, three at regional level under the ministry of local government, two at TFELTP as faculty, one at Ministry of Health Zanzibar, one at Ministry of Defence, and one at an international NGO. The graduate working with the Zanzibar Ministry of Health currently heads the Epidemiology Unit. Table 2 shows the types of activities that program residents/trainees are engaged in.

**Table 2:** Tanzania Field Epidemiology and Laboratory Training Program (TFELTP) program outputs by activity

Activity	Output/No
Outbreaks investigated and responded to by the program and its trainees	34
Surveillance data bulletins	10
Surveillance systems evaluated	33
Protocol-driven research studies undertaken	33
Program or project evaluations	4
Scientific presentations at conferences	32
Publications by the trainees in peer review journals	5
Number of health workers trained through short courses	140
Number of laboratory quality improvement projects conducted	8
Number of laboratory new technologies/test kits evaluated	3

### TFELTP role in International Health Regulations 2005 Implementation in Tanzania

In 2010, the TFELTP staff and residents participated in the country assessment of the revised International Health Regulations (IHR) core capabilities, and played a key role in the development of the assessment report. Subsequently, program staff has been involved in development of the Tanzania IHR plan. The program staff and trainees have also been involved in the incorporation of the IHR 2005 into the revised Tanzania IDSR guidelines.

### Role in high-profile outbreak investigations

TFELTP residents have been involved and led investigation and response to high profile outbreaks including cholera [15,16], H1N1 [17], among others. Residents were also involved in investigating and responding to a bomb blast in February 2011 in Dar-es-salaam. This demonstrates the service that the program is providing to the country, and the public health leaders that are being developed.

## New developments within TFELTP

TFELTP staffs have developed an NCD short course curriculum for regional capacity building for chronic disease epidemiology in collaboration with CDC DPHSWD, University of Copenhagen, and NIMR. The program has identified additional field sites for NCD mentorship of trainees. In 2011, Tanzania was one of five countries selected for CDC's global NCD initiative and conducted the first NCD epidemiology, surveillance, and evaluation two-week short course for medical doctors, TFELTP residents, and other public health staff.

TFELTP is working with the Ministry of Livestock Development on the introduction of special seminars for zoonotic diseases. Based on the one-health principle, the aim of establishing the special seminars on zoonotic diseases is to build a foundation for future development of a Veterinary track which can address priority disease conditions at the animal and human interface, and improve communication and flow of surveillance information from the animal and wildlife sectors to the MOHSW.

## Future plans of the TFELTP

The program seeks to expand enrolment to meet the needs of the public health system in Tanzania. In order to do this, the program will need to expand the available classroom and field facilities, and staff. The key priorities will be to better equip the field sites, and ensure that the field supervisors’ skills are continually improved. In order to address the shortage of competent mentors and supervisors for the trainees, one approach will be to explore the use of TFELTP graduates as mentors at the field sites.

Regional and international linkages will be an important avenue to build on the program's early successes. The program will be seeking to open its doors to potential trainees from countries neighbouring Tanzania that do not have programs of their own, Burundi, Malawi, Zambia are examples. This will greatly facilitate progress to improve cross-border collaboration in infectious disease surveillance and implementation of the IHR. Additionally, the program would like to open the NCD short course for training opportunities for regional participants. TFELTP plans to take a leading role in advocacy of IHR 2005 at regional sites within Tanzania where trainees are placed.

Over the next three to four years, the program will need to establish a sustainability plan that guarantees ongoing production of high quality graduates. The plan must not only look inward at the MoHSW as the driver, funder and consumer of TFELTP products, but must also look outwards to development partners, the private sector, and trainees meeting their own tuition as the program begins to mature. This plan development process will require input from the various stakeholders to establish which parts of the program they will be willing to pay for and would find attractive to their own needs and interests. The program will need to continue periodic internal and external reviews to ensure it is meeting its mandate.

One of the immediate challenges that the program must address to assure sustainability includes development of a full range of local teaching capacity for the program. Currently the program depends on external support to teach some of the courses for which there is limited expertise within Tanzania. The program will also need to work with the Human Resource Department of the MOHSW, and other relevant stakeholders to develop a career path for graduates of the program. This career path will be critical for defining job placement and promotion within the government system, an important framework for graduate retention. Finally, the program leadership will need to build on the early success of using an all-inclusive strategy to ensure that old partners remain committed to the program, as new ones join the collaboration.

## Conclusion

The TFELTP was developed out of the Tanzania's need to address pressing public health systems and human resource gaps. As the country's lead agency responsible for health, the MOHSW has driven the development of the program and remained at the pinnacle of leadership to ensure key stakeholders are involved, and the country's needs are addressed.

Over the last 3 years, the program has made a significant contribution to development of expertise for IDSR implementation, and other MOHSW programs and strategies. TFELTP has made important contributions to the training of Tanzania's field epidemiologists and public health laboratorians. While training the future public health leaders of the country, this program simultaneously provides a much needed service by responding to acute outbreaks, evaluating and improving public health surveillance systems, and strengthening disease control programs.

The TFELTP will need to develop a clear sustainability plan that includes both internal and external support (academic and financial) to ensure that Tanzania's public health needs are addressed in the medium to long-term.
